# Using machine learning to predict health-related quality of life outcomes in patients with low grade glioma, meningioma, and acoustic neuroma

**DOI:** 10.1371/journal.pone.0267931

**Published:** 2022-05-04

**Authors:** Roshan Karri, Yi-Ping Phoebe Chen, Katharine J. Drummond

**Affiliations:** 1 Department of Neurosurgery, The Royal Melbourne Hospital, Parkville, VIC, Australia; 2 Department of Medicine, University of Melbourne, Parkville, VIC, Australia; 3 Department of Computer Science and Information Technology, La Trobe University, Bundoora, VIC, Australia; 4 Department of Surgery, University of Melbourne, Parkville, VIC, Australia; Universitat Politecnica de Catalunya, SPAIN

## Abstract

**Background:**

Predicting reduced health-related quality of life (HRQoL) after resection of a benign or low-grade brain tumour provides the opportunity for early intervention, and targeted expenditure of scarce supportive care resources. We aimed to develop, and evaluate the performance of, machine learning (ML) algorithms to predict HRQoL outcomes in this patient group.

**Methods:**

Using a large prospective dataset of HRQoL outcomes in patients surgically treated for low grade glioma, acoustic neuroma and meningioma, we investigated the capability of ML to predict a) HRQoL-impacting symptoms persisting between 12 and 60 months from tumour resection and b) a decline in global HRQoL by more than the minimum clinically important difference below a normative population mean within 12 and 60 months after resection. Ten-fold cross-validation was used to measure the area under the receiver operating characteristic curve (AUC), area under the precision-recall curve (PR-AUC), sensitivity, and specificity of models. Six ML algorithms were explored per outcome: Random Forest Classifier, Decision Tree Classifier, Logistic Regression, K Neighbours Classifier, Support Vector Machine, and Gradient Boosting Machine.

**Results:**

The final cohort included 262 patients. Outcome measures for which AUC>0.9 were Appetite loss, Constipation, Nausea and vomiting, Diarrhoea, Dyspnoea and Fatigue. AUC was between 0.8 and 0.9 for global HRQoL and Financial difficulty. Pain and Insomnia achieved AUCs below 0.8. PR-AUCs were similar overall to the AUC of each respective classifier.

**Conclusions:**

ML algorithms based on routine demographic and perioperative data show promise in their ability to predict HRQoL outcomes in patients with low grade and benign brain tumours between 12 and 60 months after surgery.

## Introduction

Advances in surgical techniques and adjuncts, and increasing adjuvant therapy options, have led to dramatic improvements in the care of brain tumour patients over the past two decades. Consequently, with extended survival and reduction of gross neurological morbidity, measures of treatment success have appropriately shifted to more patient-centred metrics, including health-related quality of life (HRQoL) [1, 2]. HRQoL is a complex, self-assessed, multidimensional concept that encompasses the physical, emotional, role, social and cognitive components of quality of life (QoL) associated with illness and its treatment. Whilst by its nature, HRQoL data is subjective and thus intuitively assessed by qualitative measures, the introduction of structured, validated questionnaires has ensured an excellent level of consistency in its determination. Widely validated instruments such as the European Organization for Research and Treatment of Cancer (EORTC) [3] QLQ-C30 questionnaires are an example of disease and/or demographic-specific questionnaires that have internationally standardised the assessment of HRQoL.

The utility of the QLQ-C30 and other instruments lies not only in their wide validation but in the instrument-specific normative reference data that is available. For the QLQ-C30, a large pooled European normal population database (N = 16,151) [4] provides global and domain-specific averages to which oncology patient data can be compared. Using normative reference data for a standardised questionnaire, one can derive ‘minimally clinically important differences’ (MCIDs), typically taken as a proportion of the standard deviation (SD) of the baseline HRQoL score for the population [5]. These are an indication of what degree of change in HRQoL, such as may occur post-intervention or through disease trajectory, should be deemed ‘significant’ and therefore warrant clinical attention. Importantly, there is debate as to what proportion of the standard deviation is suitable to use. With regards to the QLQ-C30 global score, Maringwa et al. [6] suggested that 0.5SD is an appropriate figure to use in a clinical context, and this is widely accepted. Evidence is less comprehensive for the determination of MCIDs for symptom scores of the QLQ-C30, however [7].

Depending on the tumour location, patients with meningioma (MN), low grade glioma (LGG), or acoustic neuroma (AN) may experience a range of specific physical, cognitive and psychiatric symptoms, including neurological deficit and epilepsy [8–10]. Moreover, these patients may suffer a range of nonspecific symptoms, including headaches, fatigue, anxiety, and sleep disturbance [11, 12]. The majority of these patients are treated with surgical resection, and a smaller proportion with radiotherapy and chemotherapy therapy as alternatives or adjuncts, depending on histopathology. All these modalities can have long-lasting effects on function and HRQoL. Therefore, both the tumour and its treatment may affect HRQoL. Even in those patients with stable tumours and without significant or discernible physical or cognitive deficits, reduction in HRQoL is now recognized [13, 14].

The rationale for predicting HRQoL outcomes lies primarily in improving the efficiency of clinical interventions and secondarily in patient education. As Foster et al. [15] made clear in their paper examining the HRQoL trajectory subsequent to colorectal cancer surgery, there is tangible benefit in “identifying who is most in need of intensive support from the point of surgery so resources can be directed accordingly.” Risk stratification, and appropriate medical and allied health resource allocation (such as early interventions) to patients at high risk of poor HRQoL is the most intuitive application of predictive tools, as is managing patient expectations. In the context of post-procedural HRQoL improvement, it is often allied health expertise and psychosocial supports that constitute the bulk of impactful interventions, which commonly have limited availability. For cancer patients, prognostic information that incorporates expected HRQoL outcomes in addition to treatment morbidity and survival time, can empower them to make informed decisions regarding their care; characterising our health professional role as ‘patient-centric’ and not purely survival focussed.

Machine learning (ML) is an application of artificial intelligence (AI) that provides systems the ability to automatically learn and improve from experience without being explicitly programmed. The primary aim is to allow computers to learn automatically without human intervention or assistance and adjust actions accordingly [16]. Ultimately, ML is a modelling strategy to let the data speak for themselves, which makes it an attractive option for characterizing and predicting complex biological phenomena that do not have *a priori* models. The benefits of ML also arise from its use of a large number of tuning parameters or weights, which control the algorithm’s complexity and are estimated from the data using numerical optimization [17].

In our literature review conducted 07/07/2021, Yang et al. [18], Tsai et al. [19], Shi et al. [20], and Kumar et al. [21] are the only reports of ML models to predict HRQoL outcomes in cancer patients, however none of these algorithms are specific to brain tumours. Thus, the use of ML to predict LGG, MN, and AN HRQoL outcomes is yet unexplored. Central nervous system tumours present unique physiological and psychosocial challenges to patients unseen in other cancer types, warranting predictive modelling specific to this demographic. Currently, no decision tool exists capable of flagging patients in this demographic who are at risk of short-medium term decline in their HRQoL. Using a large and unique database, in addition to a systematic and extensive exploration of ML approaches, we aimed to develop, and evaluate the performance of ML algorithms to predict HRQoL outcomes in this patient group. We outline the performance metrics, and model development process of 10 binary classifiers predicting either the presence/absence of symptoms, or a ‘significant’ decline in global HRQoL relative to a normative population mean [4], within 12 and 60 months after tumour resection. The nature of the unique database upon which these models were trained is also discussed, informing their potential for broader applications.

## Methods

### Study population

#### Neuro-oncology health-related quality of life database

The deidentified HRQoL database is part of a longitudinal and ongoing study of HRQoL in post-operative patients with LGG, MN and AN at the Royal Melbourne Hospital Neuro-Oncology and Neurosurgery Outpatient and Private clinics which commenced in February 2014 and is approved by the Melbourne Health Human Research Ethics Committee (study number 2013.246). It comprises demographic, clinical, and radiological patient features collected at routine follow-up appointments as well as HRQoL measures. It is the largest database of its kind worldwide. Whilst data collection is ongoing, the algorithms reported here were trained and evaluated on a static dataframe downloaded on 30/07/2019. There is no predetermined periodicity to data collection for our patient cohort; collection is incidental and based on individual follow-up frequency. Data collected includes: EORQTC QLQ-C30 and the brain tumour specific module, QLQ-BN20, as well as demographic, tumour and clinical details.

#### Inclusion criteria for predictive models

All patients were 18 years or older, and had undergone surgical resection of a LGG, MN, or AN. As the original aim of the database was to determine longitudinal, long-term HRQoL outcomes in brain tumour patients, these tumour types were chosen as each have extended post-operative survival and confer risk of seizures and temporary or persistent neurological deficit, with significant HRQoL concerns. The use of more than one tumour type increased our sample size. Despite the heterogeneity of tumour types our approach has been validated by the remarkable similarity in HRQoL outcomes in our published MN [22] and LGG [23] cohorts. This is also true for AN patients in our cohort (manuscript in preparation). These results encouraged us to combine all patients for this manuscript to provide a dataset of a size conducive to meaningful modelling. Patients with other brain or spine lesions and those with neurofibromatosis type 1 or 2 were excluded. Patients needed the ability to complete the questionnaires independently in English. Patients were approached opportunistically for participation while in the clinic waiting room, and written informed consent was obtained. Participants completed the study questionnaires before or after their scheduled appointment. In a subset of consenting patients, follow-up questionnaires are completed by mail to obtain longitudinal assessment. Patients entered the study at any point post-operative and then completed the questionnaires at every post-operative visit.

### Outcome definitions

The QLQ-C30 [24] is a 30-item questionnaire that assesses global HRQoL as well as its physical, role, emotional, social and cognitive domains. It also assesses cancer symptoms and is designed for all cancer patients. The responses are provided on either four- (1—not at all, 2—a little, 3—quite a bit, 4—very much) or seven-point (1—poor, 7—excellent) Likert scales.

#### Symptom algorithms

From the QLQ-C30 questionnaire, scaled symptom scores can be derived [25]. If the scaled symptom score for the patient equalled zero, this resulted in a label “0”, and if strictly greater than zero, the label “1” was derived. The symptom algorithms are therefore sensitive to cases that should be flagged as developing long term symptoms. This process was carried out for all nine possible symptom scores produced from the QLQ-C30 questionnaire, namely: Fatigue, Insomnia, Pain, Constipation, Diarrhoea, Financial difficulty, Appetite loss, Dyspnoea and Nausea and vomiting.

#### Global HRQoL algorithm

A ‘global HRQoL’ score is produced from the QLQ-C30 questionnaire. Our binary target variable in this case was a based on whether the global HRQoL score had fallen ≥1 MCID below the normative population mean of 75 [4]. In such cases, patients would receive a label “1”. The algorithm is therefore sensitive to patients predicted to have HRQoL lower than the normative population over time. Those patients whose global HRQoL remained above the threshold were labelled “0”. The MCID used was 10 points, in keeping with previous reports [6, 26, 27], resulting in a score of 65 for the ‘threshold’.

### Variable selection

All preoperative, intra-operative, and early post-operative variables were analysed for inclusion in the predictive modelling. Initial exploration of the data involved univariate analysis of variables, using Pearson’s Chi-Squared test for categorical variables, and Welch two-sample t-tests for continuous variables. All relevant variables were included in ML models regardless of univariate significance. The final variables included are listed in Table 1 and were consistent across all nine symptom algorithms and the global HRQoL algorithm. Correlation coefficients can be found in Appendices 1 and 2 of the S1 File.

**Table 1:** Variables included in the ML models.

Variable	Variable Type	Factor Levels
Age	Continuous	N/A
Study site	Discrete	Public
		Private
		Mailout
Sex	Discrete	Male
		Female
Relationship status	Discrete	Married
		De Facto
		Other relationship
		Single
		Divorced
		Widowed
Tumour lateralisation	Discrete	Left
		Right
		Midline/Bilateral
Histological diagnosis	Discrete	Acoustic neuroma
		Meningioma
		Astrocytoma
		Oligodendroglioma
		Oligoastrocytoma
		Ependymoma
		Mixed glioma
Histological Grade ‘v2’	Discrete	2007 four-stage WHO grading system [37] for CNS tumours. From 2016 the updated system [38] was used.
		WHO Grade I
		WHO Grade II
Maximum radiological tumour diameter (abbrev. ‘Max Diameter’).	Continuous	This was the greater value of the ‘AP’ and ‘Lateral’ radiological tumour diameters listed for each patient.
Extent of resection	Discrete	Biopsy
		Partial resection
		Subtotal resection
		Gross macroscopic resection
History of radiotherapy	Discrete	Yes
		No
History of chemotherapy	Discrete	Yes
		No
Seizure history	Discrete	Yes
		No

*WHO*: *World Health Organization*, *AP*: *Anteroposterior*.

### Data filtration and processing

The original dataset consisted of 1196 patients. Of these patients, those who had received only one surgery and whose first survey had been conducted within 12–60 months of tumour resection totalled 262. If multiple surveys were completed in this time, only the first was utilised. The dataset was limited to patients who had only one surgery due to the confounding effect on HRQoL of multiple surgeries, therefore impacting predictability. The timeframe of 12–60 months was chosen taking both clinical utility and data availability into consideration. Predicting poor HRQoL at least 12 months postoperatively, as opposed to a shorter time-frame, provides time for those patients who will return to a normal HRQoL to do so and allows ample time for patients predicted to have impaired HRQoL to be directed into available, contextually appropriate rehabilitation or support services. Furthermore, predicting symptoms or global HRQoL beyond 5 years is unlikely to be relevant, given the opportunity for confounders (such as other illnesses or life events) over this time, as well as the increased chance of loss to follow up or death. With regards to data pre-processing, information stored in ‘date’ format was utilised to calculate the duration of time intervals in months. All information specifying identity, such as subject and survey IDs, were removed in the pre-processing stage. For any missing values in the final dataframe, kNN (k-nearest neighbours) imputation was performed using R statistical software (version 3.5.3), with k = 5.

### Machine learning model development and performance evaluation

#### Model types

Six ML algorithms were explored: Random Forest Classifier (RF), Decision Tree Classifier (DT), Logistic Regression (LR), K Neighbours Classifier (KNN), Support Vector Machine (SVM), and Gradient Boosting Machine (GBM). LR is a generalised linear method of classification, that models the response variable via a logistic transformation of an affine. Tree-based algorithms split the feature space into sets, fitting a model in each one [28]⁠. RF and GBM are ensemble methods that combine the output of a collection of weaker decision tree classifiers to make a strong classifier [28]. KNNs find the ‘k’ nearest training samples in multidimensional space and classify based on the majority class of these samples. Finally, SVMs learn a decision boundary (called a hyperplane) through a high dimensional space to classify samples [28].

To address the training imbalance between the relevant binarized variables in each of the ten outcome measures, synthetic minority oversampling technique (“SMOTE") was used to optimise model training. In SMOTE, the minority class is over-sampled by taking each minority class sample and introducing synthetic examples along the line segments joining any of the k minority class nearest neighbours [29]. Our implementation utilised a k value = 5. This method has been established as appropriate to ML applications in clinical contexts [30]. Hyperparameter optimisation was achieved with grid search. Models were supplied the same input variables and area under the curve (AUC) was the main optimisation metric. Final hyper-parameter values, and training metrics are available in Appendix 3 of the S1 File. Machine learning models were constructed using open-source software libraries (Python version 3.6, scikit-learn version 0.23).

#### Training and evaluation

In order to train and test the algorithms, 10-fold cross-validation repeated once was used to assess performance. Metrics measuring performance were the AUC, sensitivity, and specificity. Additionally, area under the precision-recall curve (PR-AUC) was included as a performance metric, given the imbalanced nature of the dataset [31].

## Results

### Patient characteristics

The final patient cohort on which modelling was performed included 262 patients. The median time to survey was 29 months (range 12–60). The mean age was 51.7 years (SD 14.6), with 65.6% female participants. The distribution of tumour lateralisation was 46.5%,43.4%,10.2% for left, right and midline/bilateral respectively. The majority of patients had MN (51.0%), accounting for the female predominance, with the remainder evenly spread between AN (25.3%) and LGG (23.8%). Most participants had gross macroscopic resections (66.4%), whilst the proportion of patients who received subtotal resections, partial resections, and biopsies were 22.7%, 3.9% and 7.0% respectively. In addition to their surgical management, 11.5% of patients received radiotherapy, 3.43% received chemotherapy, and 2.67% of received both chemotherapy and radiotherapy. The median ‘maximum radiological tumour diameter’ was 2.9 cm (Range 0.4–10). Table 2 shows the binarization distribution (1 vs 0 split) for each of the 10 outcome variables present in the final dataset.

**Table 2:** Distribution of binarization for each of the 10 target outcomes in the dataset.

Variable	Total ’1’	Total ’0’	Proportion of ’1’ (Total/262)	Proportion of ’0’ (Total/262)
Global HRQoL	75	187	0.29	0.71
Appetite Loss	52	210	0.20	0.80
Constipation	37	225	0.14	0.86
Financial difficulty	82	180	0.31	0.69
Nausea and vomiting	63	199	0.24	0.76
Pain	120	142	0.46	0.54
Diarrhoea	27	235	0.10	0.90
Dyspnoea	73	189	0.28	0.72
Fatigue	191	71	0.73	0.27
Insomnia	142	120	0.54	0.46

*HRQoL*: *Health-related quality of life*.

### Machine learning model performance

The best performing ML model in every outcome measure was SVM, apart from Pain and Diarrhoea, for which RF was the best performing algorithm. This suggests that differentiation by hyperplane in multidimensional space played an influential role in predictive performance across the suite of predicted target variables. RF was only optimal in two circumstances (Pain and Diarrhoea), indicating that these outcomes were more inclined to differentiation by decision-tree based systems.

Broadly speaking, the predictive capability (as measured by AUC) of the best performing algorithms for each target variable belonged to one of three main categories: >0.9, 0.8–0.9 or <0.8. The outcome measures for which AUC>0.9 were Appetite loss, Constipation, Nausea and vomiting, Diarrhoea, Dyspnoea and Fatigue. Secondly, AUC was between 0.8 and 0.9 for Global HRQoL and Financial difficulty. Finally, Pain and Insomnia achieved comparatively poorer performance, with AUC consistently below 0.8. PR-AUCs were similar overall to the AUC of each respective classifier. Except in the case of Pain and Diarrhoea, PR-AUCs obtained higher standard deviation values over cross-validation in comparison to their AUC counterpart for a given model.

**Fig 1:**
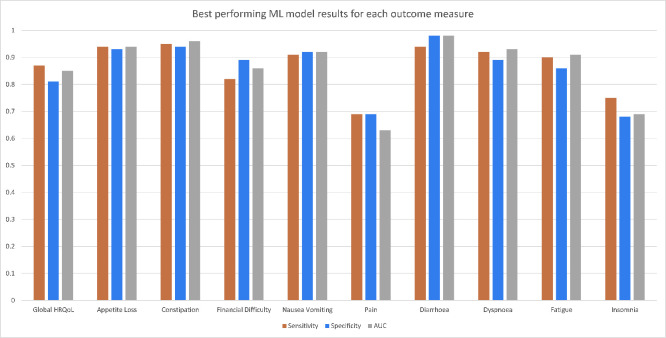
Optimal model performance metrics across the different outcome measures.

**Fig 2:**
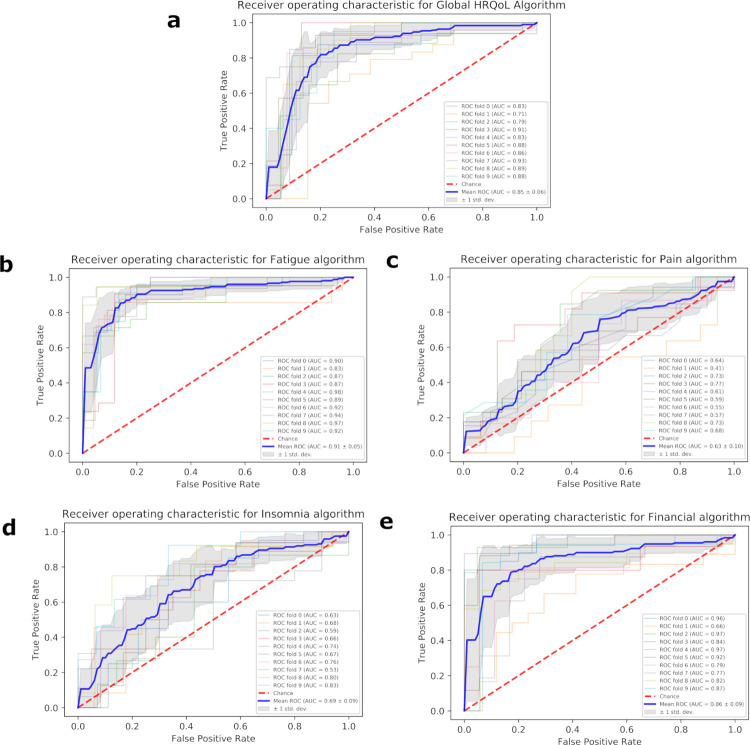
Receiver operating characteristic of the best performing algorithm for the five most prevalent outcome measures in the dataset.

**Fig 3:**
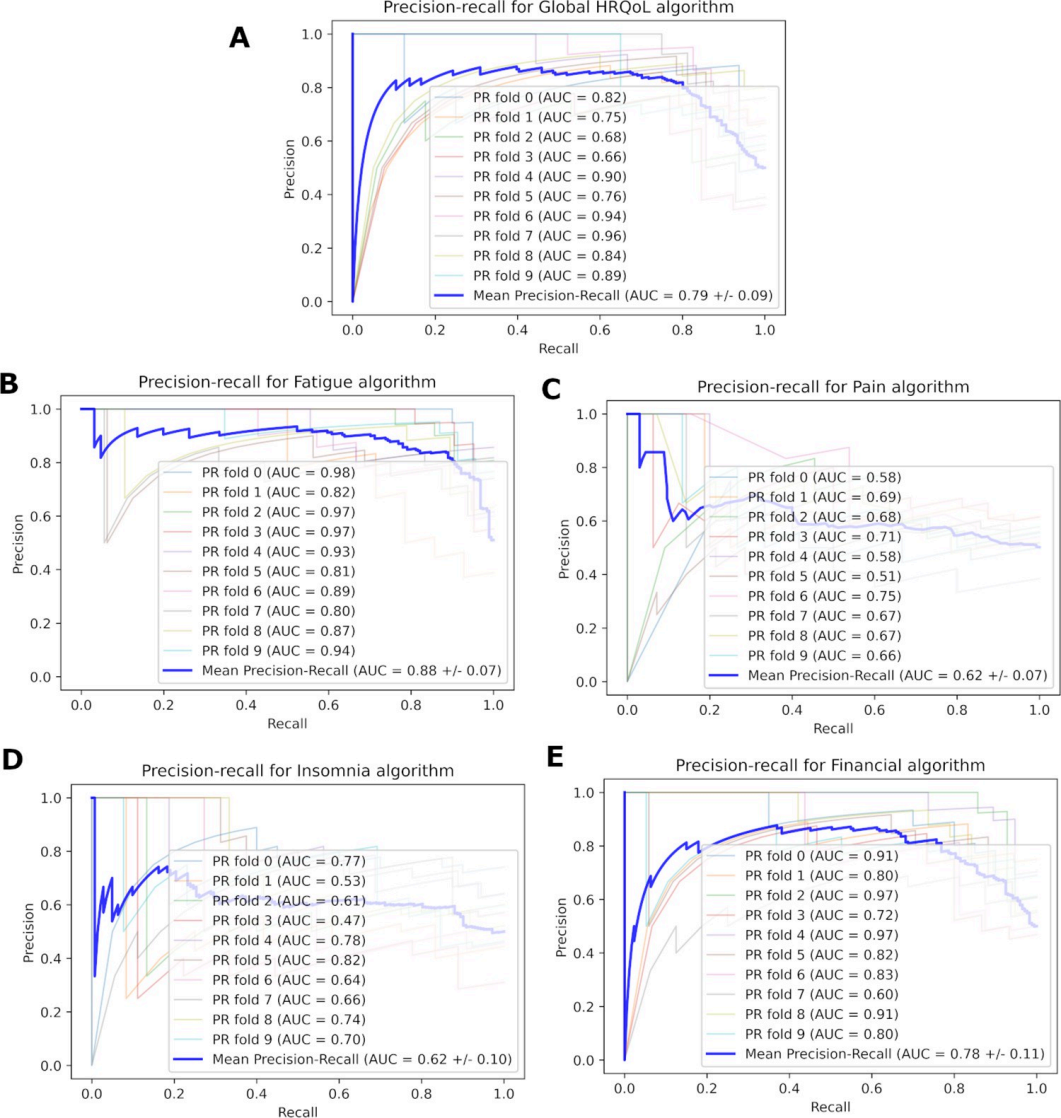
Precision-recall curve of the best performing algorithm for the five most prevalent outcome measures in the dataset.

A complete outline of the optimal ML algorithm type, AUC, PR-AUC, sensitivity and specificity for each of the outcome measures can be seen in Table 3, with head-to-head comparisons illustrated in Fig 1. Overall, the highest AUC achieved in the dataset was by SVM in relation to the Constipation target variable (0.96+/-0.03), and the lowest by RF for the Pain target variable (0.63+/-0.10). By a slight majority, ML algorithm sensitivity was generally greater or equal to specificity, as was seen in the Global HRQoL, Appetite loss, Constipation, Pain, Dyspnoea, and Fatigue models. The remaining models had a higher specificity than sensitivity. Receiver operating characteristic (ROC) and precision-recall (PR) curves for the best performing algorithm in the five most prevalent target variables (as per Table 2) can be seen in Figs 2 and 3 respectively. Remaining ROC and PR curves are illustrated in Appendices 4 and 5 of the S1 File, respectively.

**Table 3:** Optimal model performance and algorithm type for each of the ten outcome variables.

	AUC	PR-AUC	Sensitivity	Specificity	Optimal ML Algorithm
Global HRQoL	0.85+/-0.06	0.79 +/- 0.09	0.87+/-0.10	0.81+/-0.07	SVM
Appetite loss	0.94+/-0.04	0.91 +/- 0.08	0.94+/-0.06	0.93+/-0.04	SVM
Constipation	0.96+/-0.03	0.92 +/- 0.07	0.95+/-0.03	0.94+/-0.05	SVM
Financial difficulty	0.86+/-0.09	0.78 +/- 0.11	0.82+/-0.08	0.89+/-0.11	SVM
Nausea and vomiting	0.92+/-0.04	0.88 +/- 0.07	0.91+/-0.06	0.92+/-0.05	SVM
Pain	0.63+/-0.10	0.62 +/- 0.07	0.69+/-0.15	0.69+/-0.19	RF
Diarrhoea	0.98+/-0.02	0.98 +/- 0.01	0.94+/-0.05	0.98+/-0.05	RF
Dyspnoea	0.93+/-0.03	0.88 +/- 0.05	0.92+/-0.08	0.89+/-0.06	SVM
Fatigue	0.91+/-0.05	0.88 +/- 0.07	0.90+/-0.04	0.86+/-0.06	SVM
Insomnia	0.69+/-0.09	0.62 +/- 0.10	0.75+/-0.18	0.68+/-0.16	SVM

*HRQoL*: *Health-related quality of life*, *AUC*: *Area under the receiver operating characteristic (ROC) curve*, *PR-AUC*: *Area under the precision-recall (PR) curve*, *ML*: *machine learning*, *SVM*: *Support Vector Machine*, *RF*: *Random Forest Classifier*.

## Discussion

To the best of our knowledge, this is the first study to show that ML algorithms may predict HRQoL outcomes in postoperative primary brain tumour patients, specifically LGG, MN, and AN.

### Model performance and limitations

Given that the ML models in this report are the first for HRQoL determination in the three aforementioned brain tumour types, no tumour-specific baseline exists to which the performance of our models can be compared. Global HRQoL algorithms have been developed to predict HRQoL in breast cancer patients with a reported AUC of 0.90 [20] and in cervical cancer patients, with an AUC of 0.84 [21]. This is comparable to the best performing ML model for global HRQoL in this investigation, which achieved an AUC of 0.85 +/- 0.06.

Although our dataset is the largest of its kind in the world, the tumours in question are rare, which influenced our decision to analyse both benign tumours and low-grade gliomas collectively (maximising the sample available for model training). Each of these tumours have extended post-operative survival but with risk of seizures and temporary or persistent neurological deficit, with significant HRQoL concerns. Our approach has been validated by the remarkable similarity in HRQoL outcomes in our research group’s meningioma [22] and low grade glioma [23] cohorts, with fatigue, sleep disturbance and perceived cognitive deficit topping the list of concerns for both tumour types. Nevertheless, there is still some heterogeneity of symptom burden experienced by patients with different tumour types (26), especially for symptoms such as fatigue and insomnia. We hypothesize that stratification by tumour type is likely to boost performance in future modelling, and will prove more practical with ongoing data collection.

The majority of models described in this study are accurate, generalizable, and ready to be deployed for the purposes of stratifying patients at risk of HRQoL decline to enable hospital resource rationalisation. That being said, it is recognised that as the number of potential risk factors increases, the complexity of ML models can cause over-fitting [32]. Although our investigation attempted to address the issues of overfitting via active and appropriate choice of pre-training and hyper-parameter selection [33], ML models for global HRQoL, Pain, Insomnia, Financial difficulty and Fatigue outcome measures suffered from overfitting as indicated by performance discrepancies in the training and test sets during cross-validation (see Appendix 3 of the S1 File). This may suggest that the algorithms for these outcomes are less likely to translate well in external validation, and that they will benefit in particular from ongoing data collection and additional model training.

### Clinical implications

From a health-economics standpoint, low-cost interventions such as online tools have shown mixed results for improvement of HRQoL trajectories [34], meaning that there is a continued reliance on more expensive support services such as those provided by allied health practitioners in hospitals, including comprehensive multidisciplinary rehabilitation programs catering to fatigue management, cognitive rehabilitation and psychological support [35, 36]. Thus, there is an imperative to ensure that these resources are being used as efficiently as possible.

**Fig 4:**
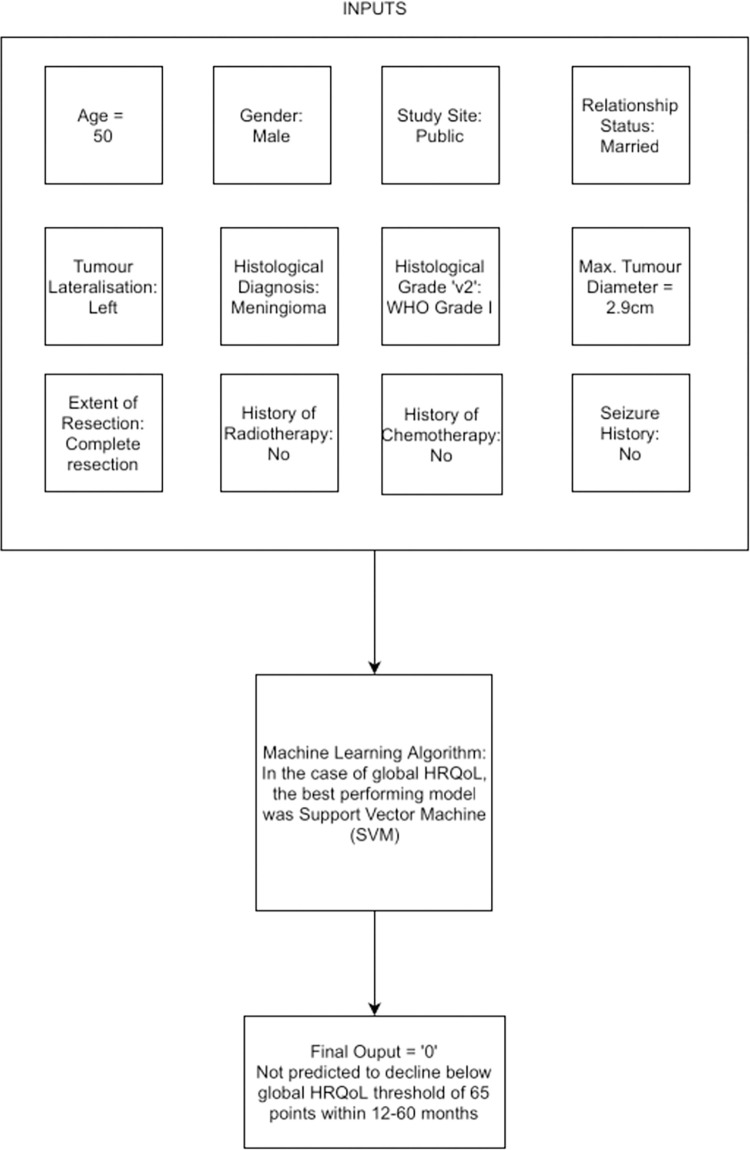
Worked example of ML model for global HRQoL. Twelve demographic and perioperative data inputs are used by the Support Vector Machine to predict whether global HRQoL will decline below the normative population mean (4) within 12–60 months of tumour resection.

The real-world application of these ML algorithms lies in their ability to predict, in the immediate post-operative period, whether a patient is likely to have poor global HRQoL or high symptom burden between 12 and 60 months of tumour resection, and by extension, those whose symptoms will have resolved within 12 months and whose HRQoL has returned to normal. Those who are identified at risk, can be streamlined early into appropriate rehabilitation and supportive care services in a cost-effective and patient-centric way. Fig 4 illustrates the process by which our SVM algorithm would make a prediction on an example patient.

An important barrier to the uptake of ML models, however, is the nature of the input variables that drive them. If inputs are resource-intensive to ascertain (for example, some imaging modalities), this restricts the implementation of the predictive systems in low resource or time poor settings. There is value in ‘future-proofing’ the ML predictive systems developed in the current health-policy climate by tailoring the inputs used. Future investigations may focus on ‘low-resource’ algorithms that do not require specialist expertise to acquire input variables. Our final models utilise input variables that are mostly not resource restricted, and therefore likely to be validated in both tertiary and non-tertiary clinical contexts in the future. The only exception is ‘maximum radiological tumour diameter’ which requires a radiologist to ensure accurate delineation of the tumour boundaries.

### Limitations conferred by the dataset

As well as the degree of overfitting, clinical utility of deployable ML algorithms is additionally defined by the generalisability of the derivation cohort on which the models are built. We accept that this is a single centre study, and that recruitment of additional intra- and inter-state centres is required to aid wide model applicability. That being said, The Royal Melbourne Hospital Neurosurgery Outpatients is a tertiary/quaternary referral service, and includes both public (government insurance) and private (insured) patients from rural, urban and remote populations, and the only major exclusions are terminal or functionally impaired patients who cannot attend clinic visits. The patient population is managed by more than 16 different neurosurgeons and a large multidisciplinary team across three partner hospitals. Thus, there is not likely to be significant selection bias in the population that is approached for data collection or in the HRQoL database derived.

The nature and timing of the questionnaires employed cannot account for post-traumatic growth, a poorly acknowledged factor contributing to improved HRQoL over time. This phenomenon refers to psychological mechanisms that enable patients to cope with trauma and that lead to positive mental change to accept the “new normal” [37, 38]. The ML models developed in this study are trained exclusively on questionnaire-derived data as the target variable, therefore the HRQoL predictions made are limited by this phenomenon.

Patients were allowed to enter the study at any time point postoperatively as they presented for follow-up. This introduces a significant spread of initial responses and potential data heterogeneity. However, it also allows inclusion of all patients followed in the service for a large “real-world” cohort. As data collection continues, this heterogeneity will be progressively eroded.

Although patients who undergo biopsies may experience different long-term trends in HRQoL relative to more involved procedures, they were still included in the dataset for modelling. The rationale for this was procedure type is only one of a very large number of inputs that can affect long term HRQoL in brain tumours, amongst a whole host of demographic, psychosocial and clinical factors, including the effect of the tumour itself; resected or unresected. Crucially, the algorithms in this investigation use “extent of resection” as an input (covariate), so are therefore trained to profile the risk of HRQoL decline for biopsies vs resections differently (and accurately). Recent expert reviews [39] in this space support the inclusion of procedure type (biopsy vs resection) in the prognostication of post-surgical outcomes in glioma. Given this rationale, and the goal to implement a practical tool in a real-world neurosurgical outpatient setting, biopsy patients were not modelled separately.

Finally, there are no preoperative baseline data. These would, of course, be interesting data but were not included for a number of reasons. The primary reason was that the original study from which this database is derived did not aim to assess the effect of treatment on HRQoL, but rather to determine factors influencing HRQoL in a large group of postoperative patients, to identify interventions for improvement, given that treatment could not be avoided [22]. In addition, the difficulty of interpreting HRQoL measures in preoperative patients recently confronted with the diagnosis of a brain tumour brings its own complexities. The assertion that pre-operative measurements of HRQoL is a true baseline in the context of a patient who has just received a diagnosis of a brain tumour is tenuous given the influence of the diagnosis on psychosocial and physical context, particularly if the patient presents with seizures or neurological deficits. The required true baseline would, in fact, be “pre-diagnosis” testing, which is not feasible. Additionally, changes after surgical treatment may not be related to the effects of treatment alone, but include a feeling of relief at “successful” treatment [22]. This investigation addressed the need for baseline data by using the normative population mean and established MCID [4, 6] as an external reference point for global HRQoL. Admittedly, a large pooled European database was used given the absence of Australian data. The applicability of future algorithms will benefit from demographic-specific baseline data. For the symptom score algorithms, an arbitrary 0 vs non-0 binarization threshold was utilised in lieu of a lack of concrete evidence surrounding symptom-specific MCID [7]. Predicting the presence or absence of symptoms may be of greater clinical utility with respect to streamlining of patients into appropriate rehabilitation streams, compared to predicting symptom score variation of a particular magnitude. Future investigations may alter these binarization criteria as a means to optimize ML algorithm predictive performance.

## Conclusion

ML algorithms based on routine demographic and perioperative data show promise in their ability to predict HRQoL outcomes in patients with low grade and benign brain tumours between 12 and 60 months after surgery. This includes both global HRQoL, as defined by the EORTC QLQ-C30, as well as symptom-specific metrics derived from the questionnaire. These models have the potential to predict patients likely to suffer poor HRQoL trajectories, and therefore inform effective allocation of sparse, resource intensive rehabilitation and supportive care services. These models, however, are limited by the small sample, single centre dataset upon which they are derived. Further data collection, ideally at a multi-centre scale, is required to ensure improved generalisability of the algorithms, as well as enabling development on tumour-stratified data to account for symptom heterogeneity between tumour types.

## Supporting information

S1 File (PDF)

S1 Dataset (CSV)
