# Supplementary material for: Using machine learning to predict health-related quality of life outcomes in patients with low grade glioma, meningioma, and acoustic neuroma
Source: PLoS One. 2022 May 4;17(5):e0267931. doi: 10.1371/journal.pone.0267931 (PMC9067699; doi:10.1371/journal.pone.0267931)

Supplementary Materials (S1)

Appendix 1 - Correlation coefficients for discrete variables

	Variable	statistic	p.value	paramete	method
	Study Site	0.003767465	0.951057	1	Pearson's Chi-squared test with Yates' continuity correction
	Gender	0.006612614	0.935189	1	Pearson's Chi-squared test with Yates' continuity correction
	Relationship Status	1.987773048	0.850834	5	Pearson's Chi-squared test
	Tumour Lateralisation	2.618391168	0.270037	2	Pearson's Chi-squared test
Global HRQoL	Histological Diagnosis	4.847359145	0.563534	6	Pearson's Chi-squared test
	Histological Grade v2	0.011122425	0.916009	1	Pearson's Chi-squared test
	Radiotherapy	0.060024486	0.806457	1	Pearson's Chi-squared test with Yates' continuity correction
	Chemotherapy	0.414328885	0.51978	1	Pearson's Chi-squared test with Yates' continuity correction
	Seizure History	0.432107511	0.805692	2	Pearson's Chi-squared test
	Extent of Resection	2.491888849	0.476759	3	Pearson's Chi-squared test
	Variable	statistic	p.value	paramete	method
	Study Site	0.541311326	0.46189	1	Pearson's Chi-squared test with Yates' continuity correction
	Gender	0.223211538	0.636604	1	Pearson's Chi-squared test with Yates' continuity correction
	Relationship Status	9.945187024	0.076803	5	Pearson's Chi-squared test
	Tumour Lateralisation	2.4906139	0.287853	2	Pearson's Chi-squared test
Appetite Loss	Histological Diagnosis	5.875453987	0.437286	6	Pearson's Chi-squared test
	Histological Grade v2	1.11754016	0.290449	1	Pearson's Chi-squared test
	Radiotherapy	0.088776525	0.765738	1	Pearson's Chi-squared test with Yates' continuity correction
	Chemotherapy	0.313474766	0.575556	1	Pearson's Chi-squared test with Yates' continuity correction
	Seizure History	4.972715761	0.083212	2	Pearson's Chi-squared test
	Extent of Resection	5.087521723	0.165499	3	Pearson's Chi-squared test
	Variable	statistic	p.value	paramete	method
	Study Site	0.034896313	0.851813	1	Pearson's Chi-squared test with Yates' continuity correction
	Gender	3.88291748	0.04878	1	Pearson's Chi-squared test with Yates' continuity correction
	Relationship Status	5.951034041	0.311013	5	Pearson's Chi-squared test
	Tumour Lateralisation	0.200867312	0.904445	2	Pearson's Chi-squared test
Constipation	Histological Diagnosis	4.355115488	0.628737	6	Pearson's Chi-squared test
	Histological Grade v2	0.493891436	0.482197	1	Pearson's Chi-squared test
	Radiotherapy	1.403933176	0.236066	1	Pearson's Chi-squared test with Yates' continuity correction
	Chemotherapy	1.323972426	0.24988	1	Pearson's Chi-squared test with Yates' continuity correction
	Seizure History	1.419594633	0.491744	2	Pearson's Chi-squared test
	Extent of Resection	5.635682345	0.130745	3	Pearson's Chi-squared test
	Variable	statistic	p.value	paramete	method
	Study Site	0	1	1	Pearson's Chi-squared test with Yates' continuity correction
	Gender	0.00324411	0.954579	1	Pearson's Chi-squared test with Yates' continuity correction
	Relationship Status	6.969976215	0.222883	5	Pearson's Chi-squared test
	Tumour Lateralisation	0.410489253	0.814448	2	Pearson's Chi-squared test
Financial	Histological Diagnosis	11.6114561	0.07122	6	Pearson's Chi-squared test
	Histological Grade v2	8.484238918	0.003582	1	Pearson's Chi-squared test
	Radiotherapy	0.59453863	0.440669	1	Pearson's Chi-squared test with Yates' continuity correction
	Chemotherapy	0.19380259	0.65977	1	Pearson's Chi-squared test with Yates' continuity correction
	Seizure History	8.924094528	0.011539	2	Pearson's Chi-squared test
	Extent of Resection	8.806946625	0.031971	3	Pearson's Chi-squared test
	Variable	statistic	p.value	paramete	method
	Study Site	0.013986664	0.905858	1	Pearson's Chi-squared test with Yates' continuity correction
	Gender	0.979907259	0.322222	1	Pearson's Chi-squared test with Yates' continuity correction
	Relationship Status	4.144383794	0.528821	5	Pearson's Chi-squared test
	Tumour Lateralisation	0.642059324	0.725402	2	Pearson's Chi-squared test
Nausea Vomiting	Histological Diagnosis	3.371422515	0.76099	6	Pearson's Chi-squared test
	Histological Grade v2	3.018594312	0.082315	1	Pearson's Chi-squared test
	Radiotherapy	0.240503059	0.623843	1	Pearson's Chi-squared test with Yates' continuity correction
	Chemotherapy	3.208671331	0.073249	1	Pearson's Chi-squared test with Yates' continuity correction
	Seizure History	2.609651707	0.27122	2	Pearson's Chi-squared test
	Extent of Resection	3.355811554	0.33995	3	Pearson's Chi-squared test
	Variable	statistic	p.value	paramete	method
	Study Site	1.958873328	0.161634	1	Pearson's Chi-squared test with Yates' continuity correction
	Gender	2.30E-30	1	1	Pearson's Chi-squared test with Yates' continuity correction
	Relationship Status	17.60033173	0.003491	5	Pearson's Chi-squared test
	Tumour Lateralisation	1.915790132	0.3837	2	Pearson's Chi-squared test
Pain	Histological Diagnosis	1.837182386	0.934047	6	Pearson's Chi-squared test
	Histological Grade v2	0.296684119	0.585969	1	Pearson's Chi-squared test
	Radiotherapy	0.860625	0.353564	1	Pearson's Chi-squared test with Yates' continuity correction
	Chemotherapy	0.032393293	0.857167	1	Pearson's Chi-squared test with Yates' continuity correction
	Seizure History	0.892785973	0.639932	2	Pearson's Chi-squared test
	Extent of Resection	4.140900383	0.246646	3	Pearson's Chi-squared test
	Variable	statistic	p.value	paramete	method
	Study Site	0.594797595	0.44057	1	Pearson's Chi-squared test with Yates' continuity correction
	Gender	2.671248785	0.102176	1	Pearson's Chi-squared test with Yates' continuity correction
	Relationship Status	3.335571065	0.6484	5	Pearson's Chi-squared test
	Tumour Lateralisation	0.534411728	0.765515	2	Pearson's Chi-squared test
Diarrhoea	Histological Diagnosis	1.015750245	0.985008	6	Pearson's Chi-squared test
	Histological Grade v2	0.132849098	0.715496	1	Pearson's Chi-squared test
	Radiotherapy	0.041768194	0.838062	1	Pearson's Chi-squared test with Yates' continuity correction
	Chemotherapy	2.911776038	0.087935	1	Pearson's Chi-squared test with Yates' continuity correction
	Seizure History	2.58523914	0.274551	2	Pearson's Chi-squared test
	Extent of Resection	2.176612108	0.53657	3	Pearson's Chi-squared test
	Variable	statistic	p.value	paramete	method
	Study Site	6.252856661	0.012399	1	Pearson's Chi-squared test with Yates' continuity correction
	Gender	3.803289411	0.051152	1	Pearson's Chi-squared test with Yates' continuity correction
	Relationship Status	11.07028049	0.050004	5	Pearson's Chi-squared test
	Tumour Lateralisation	0.294347475	0.863144	2	Pearson's Chi-squared test
Dysnpnoea	Histological Diagnosis	3.608945212	0.729423	6	Pearson's Chi-squared test
	Histological Grade v2	1.381459852	0.239853	1	Pearson's Chi-squared test
	Radiotherapy	3.90E-30	1	1	Pearson's Chi-squared test with Yates' continuity correction
	Chemotherapy	2.085673577	0.148687	1	Pearson's Chi-squared test with Yates' continuity correction
	Seizure History	0.744493982	0.689184	2	Pearson's Chi-squared test
	Extent of Resection	2.136615156	0.544541	3	Pearson's Chi-squared test
	Variable	statistic	p.value	paramete	method
	Study Site	0.108282688	0.742108	1	Pearson's Chi-squared test with Yates' continuity correction
	Gender	2.705207095	0.100021	1	Pearson's Chi-squared test with Yates' continuity correction
	Relationship Status	6.81483913	0.234779	5	Pearson's Chi-squared test
	Tumour Lateralisation	0.651855609	0.721857	2	Pearson's Chi-squared test
Fatigue	Histological Diagnosis	5.697162373	0.457954	6	Pearson's Chi-squared test
	Histological Grade v2	7.213727266	0.007235	1	Pearson's Chi-squared test
	Radiotherapy	5.083207215	0.024159	1	Pearson's Chi-squared test with Yates' continuity correction
	Chemotherapy	0.352785853	0.55254	1	Pearson's Chi-squared test with Yates' continuity correction
	Seizure History	3.670688555	0.159559	2	Pearson's Chi-squared test
	Extent of Resection	5.652863502	0.129776	3	Pearson's Chi-squared test
	Variable	statistic	p.value	paramete	method
	Study Site	0.017939087	0.893452	1	Pearson's Chi-squared test with Yates' continuity correction
	Gender	4.67282857	0.030643	1	Pearson's Chi-squared test with Yates' continuity correction
	Relationship Status	2.913470048	0.713324	5	Pearson's Chi-squared test
	Tumour Lateralisation	1.468240755	0.479927	2	Pearson's Chi-squared test
Insomnia	Histological Diagnosis	10.87736844	0.092241	6	Pearson's Chi-squared test
	Histological Grade v2	3.165716376	0.075199	1	Pearson's Chi-squared test
	Radiotherapy	0.102032478	0.749404	1	Pearson's Chi-squared test with Yates' continuity correction
	Chemotherapy	3.70522E-29	1	1	Pearson's Chi-squared test with Yates' continuity correction
	Seizure History	4.288468871	0.117158	2	Pearson's Chi-squared test
	Extent of Resection	11.43424331	0.009595	3	Pearson's Chi-squared test

Appendix 2 - Correlation coefficients for continuous variables

	Variable	estimate	estimate1	estimate2	statistic	p.value	parameter	conf.low	conf.high	method	alternative
**Global HRQoL**	Age	-4.850828729	50.14917127	55	-2.447840398	0.015611715	139.6938716	-8.768785232	-0.932872227	Welch Two Sample t-test	two.sided
	Max Diameter	0.349731459	3.293479456	2.943747997	1.752803021	0.081316936	182.2915901	-0.043947795	0.743410712	Welch Two Sample t-test	two.sided
	**Variable**	**estimate**	**estimate1**	**estimate2**	**statistic**	**p.value**	**parameter**	**conf.low**	**conf.high**	**method**	**alternative**
**Appetite Loss**	Age	0.630068208	51.61083744	50.98076923	0.26105366	0.79477858	73.76520169	-4.179305894	5.439442309	Welch Two Sample t-test	two.sided
	Max Diameter	0.21941068	3.239647987	3.020237307	1.04382424	0.298904556	107.6747715	-0.197254212	0.636075572	Welch Two Sample t-test	two.sided
	**Variable**	**estimate**	**estimate1**	**estimate2**	**statistic**	**p.value**	**parameter**	**conf.low**	**conf.high**	**method**	**alternative**
**Constipation**	Age	-7.118026283	50.44954128	57.56756757	-2.842105696	0.006473875	49.93145958	-12.14861429	-2.087438279	Welch Two Sample t-test	two.sided
	Max Diameter	0.085769681	3.207350429	3.121580748	0.360915436	0.719422322	60.38232074	-0.389528821	0.561068183	Welch Two Sample t-test	two.sided
	**Variable**	**estimate**	**estimate1**	**estimate2**	**statistic**	**p.value**	**parameter**	**conf.low**	**conf.high**	**method**	**alternative**
**Financial**	Age	5.330678133	53.19653179	47.86585366	2.849785045	0.004906639	172.9416694	1.63862258	9.022733687	Welch Two Sample t-test	two.sided
	Max Diameter	-0.104756003	3.161219173	3.265975176	-0.487011349	0.626882356	168.7290395	-0.529389365	0.31987736	Welch Two Sample t-test	two.sided
	**Variable**	**estimate**	**estimate1**	**estimate2**	**statistic**	**p.value**	**parameter**	**conf.low**	**conf.high**	**method**	**alternative**
**Nausea Vomiting**	Age	-0.898313492	51.26041667	52.15873016	-0.434957816	0.664438738	110.84241	-4.990885175	3.19425819	Welch Two Sample t-test	two.sided
	Max Diameter	0.307058512	3.270766932	2.963708419	1.481498613	0.140752928	138.0352525	-0.102760645	0.716877669	Welch Two Sample t-test	two.sided
	**Variable**	**estimate**	**estimate1**	**estimate2**	**statistic**	**p.value**	**parameter**	**conf.low**	**conf.high**	**method**	**alternative**
**Pain**	Age	-4.708333333	49.26666667	53.975	-2.604610622	0.009746215	251.1434759	-8.268500636	-1.148166031	Welch Two Sample t-test	two.sided
	Max Diameter	0.446489525	3.405018134	2.958528609	2.218844168	0.027399418	248.9946082	0.050167113	0.842811937	Welch Two Sample t-test	two.sided
	**Variable**	**estimate**	**estimate1**	**estimate2**	**statistic**	**p.value**	**parameter**	**conf.low**	**conf.high**	**method**	**alternative**
**Diarrhoea**	Age	-4.22417154	51.03508772	55.25925926	-1.343462655	0.188708245	31.54279405	-10.63242547	2.184082387	Welch Two Sample t-test	two.sided
	Max Diameter	0.24014157	3.220332171	2.980190601	0.909455508	0.368807418	38.2339071	-0.294291988	0.774575127	Welch Two Sample t-test	two.sided
	**Variable**	**estimate**	**estimate1**	**estimate2**	**statistic**	**p.value**	**parameter**	**conf.low**	**conf.high**	**method**	**alternative**
**Dysnpnoea**	Age	-6.425636008	49.64285714	56.06849315	-3.129748458	0.002181359	124.1050255	-10.48923016	-2.362041854	Welch Two Sample t-test	two.sided
	Max Diameter	0.108968416	3.226100297	3.117131881	0.537406253	0.591676887	173.1772701	-0.291244987	0.509181819	Welch Two Sample t-test	two.sided
	**Variable**	**estimate**	**estimate1**	**estimate2**	**statistic**	**p.value**	**parameter**	**conf.low**	**conf.high**	**method**	**alternative**
**Fatigue**	Age	0.419911649	51.796875	51.37696335	0.218927019	0.827049664	130.2177415	-3.374649347	4.214472645	Welch Two Sample t-test	two.sided
	Max Diameter	-0.035897233	3.168017686	3.203914918	-0.141843212	0.887496277	97.58485014	-0.538146702	0.466352236	Welch Two Sample t-test	two.sided
	**Variable**	**estimate**	**estimate1**	**estimate2**	**statistic**	**p.value**	**parameter**	**conf.low**	**conf.high**	**method**	**alternative**
**Insomnia**	Age	1.27899061	52.39166667	51.11267606	0.703273358	0.482534031	252.2370046	-2.302634177	4.860615398	Welch Two Sample t-test	two.sided
	Max Diameter	1.485397108	10.58866149	9.103264379	1.221994222	0.222878927	246.1086643	-0.908814074	3.879608289	Welch Two Sample t-test	two.sided

Appendix 3 - Hyperparameters tested, and training metrics

	Fold	fit_time	score_time	test_auc	train_auc	test_sens	train_sens	test_spec	train_spec	test_thresh	train_thresh
	0	0.007482529	0.00405	0.833333	0.992784	0.8125	0.951515	0.904762	0.9875	0.129202098	0.718731444
	1	0.006489754	0.00399	0.708333	0.999071	0.666667	0.980892	0.769231	1	0.79000652	0.717229975
	2	0.006980419	0.00399	0.786378	0.994599	0.823529	0.981707	0.736842	0.975308642	0.40464642	0.430306154
	3	0.006980896	0.002992	0.913043	0.993407	1	0.97619	0.869565	0.974683544	0.531470114	0.685397532
	4	0.005982876	0.00399	0.830247	0.988727	0.777778	0.993865	0.833333	0.969325153	0.743193012	0.00932792
Global HRQoL	5	0.006980896	0.00399	0.883117	0.998381	0.928571	0.982036	0.818182	0.974842767	0.169292556	0.565541353
SVM	6	0.005983114	0.00399	0.859532	0.993445	0.826087	1	0.923077	0.958333333	0.495663482	-0.043877836
	7	0.005983353	0.00399	0.934375	0.987578	0.9375	0.987879	0.8	0.956521739	0.062071005	0.244451751
	8	0.006980896	0.00399	0.890625	0.988331	1	0.975155	0.6875	0.96969697	-0.661479615	0.628334945
	9	0.005983114	0.00399	0.878125	0.986147	0.95	0.993789	0.75	0.963636364	0.276968222	0.460759456
	Mean	0.006582785	0.003896	0.851711	0.992247	0.872263	0.982303	0.809249	0.972984851	0.294103382	0.441620269
	SD	0.000537606	0.000302	0.062832	0.004237	0.102654	0.01284	0.071861	0.012448068	0.395832325	0.269137938
	Parameters trialled	{'C': [0.001,0.01,0.1,1,10,100,1000], 'gamma':['auto','scale']}									
	Best parameters	{'C': 10, 'gamma': 'auto'}									
	Fold	fit_time	score_time	test_auc	train_auc	test_sens	train_sens	test_spec	train_spec	test_thresh	train_thresh
	0	0.00848794	0.004993	0.913876	0.99961	0.894737	0.98913	0.909091	0.994475138	0.737957703	0.60846929
	1	0.005979776	0.00399	0.9525	0.999459	0.92	1	0.875	0.983957219	0.567789126	0.190119393
	2	0.004983187	0.00399	1	0.999519	1	1	1	0.989247312	0.397900998	0.362986128
	3	0.006014347	0.003996	0.880952	0.994445	0.952381	1	0.9	0.983606557	0.665917465	0.523443459
	4	0.006982327	0.00399	0.997608	0.99964	1	1	0.954545	0.988950276	0.512308041	0.646528158
Appetite Loss	5	0.006016016	0.003988	0.947619	0.99949	0.95	0.989071	0.952381	0.994505495	0.268196695	0.64914604
SVM	6	0.006981373	0.004988	0.868132	0.995964	0.785714	1	0.884615	0.988700565	0.570069334	0.619097026
	7	0.006013393	0.004987	0.95202	0.999283	1	0.994595	0.909091	0.988950276	0.183405407	0.357123989
	8	0.006040573	0.00399	0.949495	0.999552	0.909091	1	1	0.978378378	0.672293694	0.358003985
	9	0.005983591	0.005018	0.954887	0.999433	0.952381	0.994505	0.947368	0.989130435	0.538970308	0.575952506
	Mean	0.006348252	0.004393	0.941709	0.99864	0.93643	0.99673	0.933209	0.987990165	0.511480877	0.489086997
	SD	0.000891178	0.000493	0.041102	0.001753	0.061877	0.00436	0.042381	0.004664766	0.16973033	0.151712685
	Parameters trialled	{'C': [0.001,0.01,0.1,1,10,100,1000], 'gamma':['auto','scale']}									
	Best parameters	{'C': 10, 'gamma': 'auto'}									
	Fold	fit_time	score_time	test_auc	train_auc	test_sens	train_sens	test_spec	train_spec	test_thresh	train_thresh
	0	0.006596327	0.004535	0.937888	0.999831	0.913043	0.989744	0.904762	1	0.431561649	0.49993584
	1	0.005491257	0.004987	0.947917	0.999675	0.958333	1	0.9	0.98989899	0.247487679	0.153373683
	2	0.005984545	0.00399	0.985263	0.999818	1	0.994975	0.88	0.994818653	0.092986606	0.554706972
	3	0.005983114	0.003994	0.915289	0.997085	0.954545	1	0.863636	0.989795918	0.506001569	0.22108544
	4	0.005975723	0.00399	0.991667	0.999753	0.916667	0.994845	1	0.994949495	0.592586991	0.560709244
Constipation	5	0.005982876	0.00399	0.983437	0.996447	0.952381	0.989848	1	0.994871795	0.457842234	0.581179994
SVM	6	0.005974531	0.00399	0.984848	0.999832	0.952381	0.994924	0.909091	0.994897959	0.533618725	0.584197519
	7	0.006981373	0.003991	0.941304	0.995843	0.95	0.989899	0.956522	0.994871795	0.857631953	0.447263584
	8	0.006978989	0.003989	0.991342	0.999702	0.954545	0.989796	0.952381	0.994923858	0.612919884	0.699437428
	9	0.005983829	0.00399	0.991342	0.999702	0.909091	1	1	0.984771574	0.492281722	0.181537288
	Mean	0.006193256	0.004145	0.96703	0.998769	0.946099	0.994403	0.936639	0.993380004	0.482491901	0.448342699
	SD	0.000465271	0.000324	0.026948	0.001539	0.02573	0.004228	0.049337	0.003956818	0.195772265	0.183191476
	Parameters trialled	{'C': [0.001,0.01,0.1,1,10,100,1000], 'gamma':['auto','scale']}									
	Best parameters	{'C': 10, 'gamma': 'auto'}									
	Fold	fit_time	score_time	test_auc	train_auc	test_sens	train_sens	test_spec	train_spec	test_thresh	train_thresh
	0	0.116687059	0.011968	0.999053	0.999988	0.958333	1	1	0.995145631	0.5	0.428333333
	1	0.139626026	0.010972	1	0.999988	1	1	1	0.995238095	0.46	0.385333333
	2	0.115686178	0.009974	0.979167	1	0.875	1	1	1	0.69	0.64
	3	0.124665976	0.010971	0.946023	1	0.875	1	1	1	0.67	0.63
	4	0.1186831	0.011966	0.966346	0.999988	0.884615	1	0.95	0.995192308	0.56	0.545944444
Diarrhoea	5	0.120676994	0.010978	1	0.999988	1	0.995192	1	1	0.71	0.613333333
RF	6	0.128901958	0.011003	0.954453	0.999988	0.947368	0.995215	0.846154	1	0.22	0.6
	7	0.12070775	0.014962	0.995951	0.999988	0.947368	0.995215	1	1	0.7535	0.59
	8	0.13270545	0.011969	0.994048	0.999988	1	0.995169	0.958333	1	0.53	0.62
	9	0.117684364	0.012964	0.968379	0.999988	0.913043	1	1	0.995145631	0.7	0.518166667
	Mean	0.123602486	0.011773	0.980342	0.999991	0.940073	0.998079	0.975449	0.998072167	0.57935	0.557111111
	SD	0.007453099	0.001321	0.019347	4.75E-06	0.048465	0.002353	0.046773	0.002361229	0.153263833	0.083637988
	Parameters trialled	{'max_features': ['auto','sqrt','log2'], 'criterion':['gini','entropy'], 'n_estimators':[100,200,300]}									
	Best parameters	{'criterion': 'entropy', 'max_features': 'sqrt', 'n_estimators': 100}									
	Fold	fit_time	score_time	test_auc	train_auc	test_sens	train_sens	test_spec	train_spec	test_thresh	train_thresh
	0	0.007012367	0.004963	0.947059	0.999663	0.941176	0.993939	0.9	0.99382716	0.291149295	-0.127114766
	1	0.005983114	0.004987	0.933333	0.995734	0.818182	0.99375	1	0.994011976	0.858214982	-0.231367085
	2	0.005982876	0.00399	0.939103	0.9997	1	0.993671	0.923077	0.99408284	0.094970938	-0.095055405
	3	0.005983114	0.00399	0.872024	0.999514	0.8125	0.987952	0.904762	0.99378882	0.644477666	0.148435824
	4	0.005985022	0.004988	0.981424	0.999517	1	0.993939	0.894737	0.987730061	0.357966719	-0.078995297
Dysnpnoea	5	0.005981684	0.003992	0.944272	0.999814	0.823529	1	0.947368	0.993865031	0.500309616	-0.041723889
SVM	6	0.007977486	0.004987	0.86875	0.999777	0.95	0.993827	0.8125	0.993975904	0.871049619	0.194878174
	7	0.005983114	0.00399	0.932099	0.999628	0.888889	0.993902	0.944444	0.993902439	0.364841635	0.164753914
	8	0.005971193	0.00399	0.942857	0.999702	1	0.982036	0.857143	1	0.233494373	0.284781489
	9	0.004985571	0.003989	0.9125	0.999814	1	0.987952	0.8	1	0.044173999	0.277747225
	Mean	0.006184554	0.004387	0.927342	0.999286	0.923428	0.992097	0.898403	0.994518423	0.426064884	0.049634018
	SD	0.000750089	0.000486	0.032789	0.001189	0.07683	0.004651	0.05854	0.003297488	0.275157529	0.175347874
	Parameters trialled	{'C': [0.001,0.01,0.1,1,10,100,1000], 'gamma':['auto','scale']}									
	Best parameters	{'C': 10, 'gamma': 'auto'}									

	Fold	fit_time	score_time	test_auc	train_auc	test_sens	train_sens	test_spec	train_spec	test_thresh	train_thresh
	0	0.007054329	0.003988	0.897368	0.999286	0.9	0.994152	0.842105	0.994186047	-0.698546584	-0.419806996
	1	0.005979776	0.004989	0.828571	0.996086	0.857143	0.983051	0.88	0.987951807	-0.671025551	-0.132535339
	2	0.007018805	0.003957	0.865546	0.998952	0.857143	0.994118	0.764706	0.977011494	-0.4927887	-0.308041045
	3	0.005981207	0.004021	0.871148	0.995909	0.904762	0.982353	0.882353	1	-0.222041138	-0.335434856
	4	0.006939888	0.00399	0.983333	0.996079	0.944444	0.988439	0.95	0.988304094	-0.254669672	-0.328009826
Fatigue	5	0.005981684	0.00399	0.886364	0.999256	0.875	0.994286	0.863636	0.98816568	-0.529181979	-0.284969015
SVM	6	0.004976034	0.00399	0.915966	0.999121	0.857143	0.994118	0.882353	0.988505747	-0.673877094	-0.425857535
	7	0.004986048	0.003989	0.944444	0.996045	1	0.988439	0.75	0.98245614	-0.921746785	-0.124554458
	8	0.005984068	0.003989	0.969529	0.999358	0.947368	0.994186	0.947368	0.98255814	-0.517007404	-0.579042756
	9	0.005975723	0.002992	0.915942	0.99628	0.913043	0.988095	0.866667	0.988636364	-0.134677081	-0.361575962
	Mean	0.006087756	0.00399	0.907821	0.997637	0.905605	0.990124	0.862919	0.987777551	-0.511556199	-0.329982779
	SD	0.000709542	0.000447	0.045788	0.001563	0.044981	0.004496	0.062134	0.006030347	0.233995812	0.1280691
	Parameters trialled	{'C': [0.001,0.01,0.1,1,10,100,1000], 'gamma':['auto','scale']}									
	Best parameters	{'C': 10, 'gamma': 'auto'}									
	Fold	fit_time	score_time	test_auc	train_auc	test_sens	train_sens	test_spec	train_spec	test_thresh	train_thresh
	0	0.006614208	0.003991	0.956667	0.998138	0.8	0.960784	1	0.993670886	0.922798954	0.47779145
	1	0.005981207	0.00502	0.663399	0.998718	0.666667	0.974194	0.705882	0.993589744	0.471408771	0.720727812
	2	0.006005287	0.00399	0.965986	0.993504	0.928571	0.974843	0.952381	0.980263158	0.635843023	0.542425171
	3	0.003979206	0.004989	0.84	0.99454	0.8	0.981013	0.95	0.980392157	0.607060087	0.089263833
	4	0.005983353	0.00399	0.973684	0.993465	0.894737	0.961039	1	1	0.440714077	0.675246131
Financial	5	0.005983591	0.002993	0.915033	0.993797	0.882353	0.961538	0.944444	1	0.586636319	0.666532759
SVM	6	0.006502867	0.005029	0.788194	0.994781	0.8125	0.980892	0.777778	0.993548387	-0.462896619	0.376577727
	7	0.005987167	0.004986	0.770833	0.993959	0.875	0.961783	0.722222	1	0.486937539	0.724825057
	8	0.00598073	0.004994	0.824561	0.990301	0.684211	0.974026	0.933333	0.993670886	0.395748927	0.632381904
	9	0.005982637	0.00396	0.873684	0.993753	0.842105	0.967532	0.933333	1	0.663821596	0.632745366
	Mean	0.005900025	0.004394	0.857204	0.994496	0.818614	0.969764	0.891937	0.993513522	0.474807268	0.553851721
	SD	0.000679378	0.000671	0.094897	0.002284	0.082196	0.007791	0.106309	0.007183986	0.343396544	0.187106396
	Parameters trialled	{'C': [0.001,0.01,0.1,1,10,100,1000], 'gamma':['auto','scale']}									
	Best parameters	{'C': 10, 'gamma': 'auto'}									
	Fold	fit_time	score_time	test_auc	train_auc	test_sens	train_sens	test_spec	train_spec	test_thresh	train_thresh
	0	0.124665976	0.01895	0.65	1	0.916667	1	0.4	1	0.29	0.57
	1	0.12566185	0.01097	0.565341	1	0.6875	1	0.545455	1	0.4	0.65
	2	0.107748508	0.010963	0.846591	1	0.818182	1	0.875	1	0.52	0.62
	3	0.102732182	0.013955	0.6875	1	0.625	1	0.818182	1	0.59	0.6
	4	0.107750416	0.011967	0.641176	1	0.4	1	1	1	0.8	0.63
Pain	5	0.107749224	0.01097	0.576923	1	0.857143	1	0.384615	1	0.38	0.58
RF	6	0.104899883	0.01097	0.744505	1	0.692308	1	0.785714	1	0.52	0.6
	7	0.115694761	0.012991	0.631868	1	0.714286	1	0.692308	1	0.55	0.62
	8	0.132644176	0.011967	0.612637	1	0.5	1	0.769231	1	0.54	0.65
	9	0.104717731	0.011968	0.561111	1	0.666667	1	0.583333	1	0.5	0.68
	Mean	0.113426471	0.012567	0.651765	1	0.687775	1	0.685384	1	0.509	0.62
	SD	0.010050358	0.002327	0.08436	0	0.14875	0	0.192774	0	0.130648383	0.032249031
	Parameters trialled	{'max_features': ['auto','sqrt','log2'], 'criterion':['gini','entropy'], 'n_estimators':[100,200,300]}									
	Best parameters	{'criterion': 'entropy', 'max_features': 'auto', 'n_estimators': 100}									
	Fold	fit_time	score_time	test_auc	train_auc	test_sens	train_sens	test_spec	train_spec	test_thresh	train_thresh
	0	0.006020069	0.004953	0.955263	0.998841	0.95	0.982558	0.947368	0.988439306	0.536397765	0.399839471
	1	0.004985332	0.005023	0.944444	0.995967	0.904762	0.982456	0.944444	0.988505747	0.594220984	0.363409518
	2	0.005023003	0.004951	0.917143	0.996165	1	0.988024	0.857143	0.994382022	0.043872287	0.275105705
	3	0.005982399	0.003992	0.909091	0.995402	0.909091	0.994475	0.928571	0.975609756	0.678133683	0.353330831
	4	0.005983829	0.00399	0.933333	0.998847	0.888889	0.982759	0.95	0.994186047	0.687938027	0.477511607
Nausea Vomiting	5	0.005983353	0.004987	0.963585	0.996675	0.941176	0.988571	0.952381	0.98245614	0.807403041	0.126130234
SVM	6	0.005967855	0.00399	0.980392	0.996642	0.904762	0.988304	1	0.977142857	0.79343605	0.041323135
	7	0.004985809	0.003994	0.871148	0.993935	0.904762	1	0.882353	0.982857143	0.405476958	0.361746531
	8	0.005177975	0.004988	0.901961	0.996608	0.904762	0.988304	0.882353	0.988571429	0.70840686	0.665968831
	9	0.005983591	0.006992	0.862745	0.999014	0.764706	0.988571	0.857143	0.988304094	0.114206927	0.357164536
	Mean	0.005609322	0.004786	0.923911	0.99681	0.907291	0.988402	0.920176	0.986045454	0.536949258	0.34215304
	SD	0.000465257	0.000871	0.036783	0.001568	0.056802	0.005225	0.045282	0.006084749	0.255628711	0.164119527
	Parameters trialled	{'C': [0.001,0.01,0.1,1,10,100,1000], 'gamma':['auto','scale']}									
	Best parameters	{'C': 10, 'gamma': 'auto'}									
	Fold	fit_time	score_time	test_auc	train_auc	test_sens	train_sens	test_spec	train_spec	test_thresh	train_thresh
	0	0.005983353	0.004988	0.627778	0.785221	0.85	0.729508	0.444444	0.736842105	-0.443022199	-0.056769295
	1	0.003988504	0.004987	0.681373	0.790031	0.75	0.769231	0.705882	0.72	0.116398574	0.046994199
	2	0.004985809	0.002993	0.585714	0.803273	0.8	0.889764	0.571429	0.6171875	-0.435069152	-0.312605386
	3	0.003987312	0.00399	0.661111	0.778689	0.777778	0.842105	0.55	0.62295082	-0.207541705	-0.131218247
	4	0.003988743	0.00399	0.739583	0.77442	0.75	0.650794	0.75	0.792307692	0.262841394	0.101241473
Insomnia	5	0.003988028	0.002993	0.666667	0.777411	0.8125	0.849206	0.583333	0.630769231	-0.171757017	-0.378389088
SVM	6	0.004986286	0.00399	0.764706	0.781924	0.818182	0.801527	0.647059	0.688	0.066135679	-0.126069062
	7	0.004986286	0.003989	0.527778	0.797837	0.222222	0.814516	1	0.666666667	0.334613373	-0.16209923
	8	0.004989624	0.004021	0.802083	0.783455	0.75	0.853846	0.875	0.603174603	0.199846991	-0.288393832
	9	0.004024029	0.003994	0.825641	0.780321	0.923077	0.790698	0.666667	0.692913386	0.226977852	-0.103667947
	Mean	0.004590797	0.003994	0.688243	0.785258	0.745376	0.799119	0.679381	0.6770812	-0.005057621	-0.141097641
	SD	0.000658871	0.000631	0.09027	0.008755	0.181691	0.066046	0.155168	0.05770027	0.273551476	0.145190176
	Parameters trialled	{'C': [0.001,0.01,0.1,1,10,100,1000], 'gamma':['auto','scale']}									
	Best parameters	{'C': 100, 'gamma': 'scale'}									

Appendix 4 – Receiver operating characteristic of the best performing algorithm for the 5 least prevalent target variables

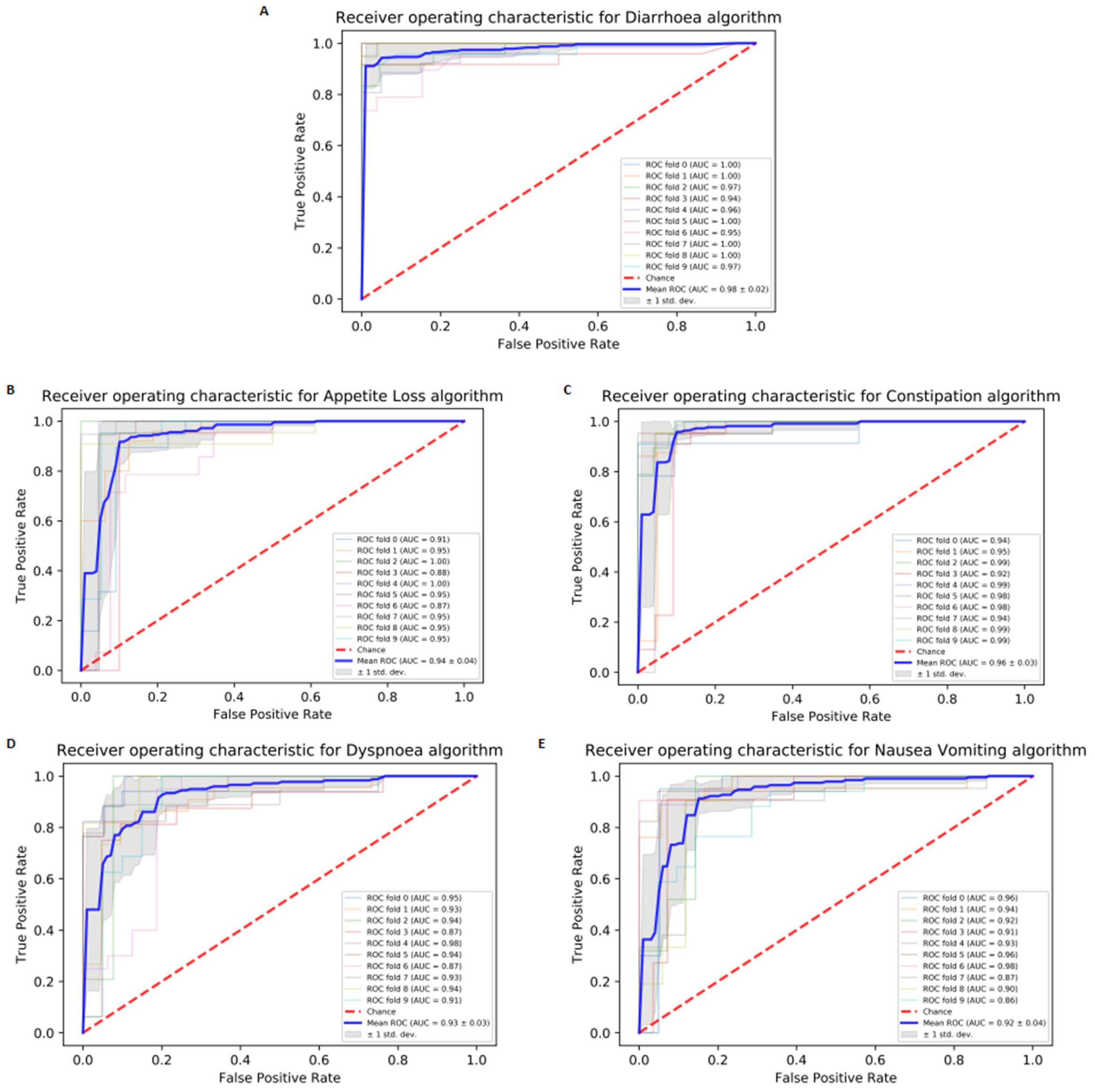

Appendix 5 – Precision-recall curve of the best performing algorithm for the 5 least prevalent target variables

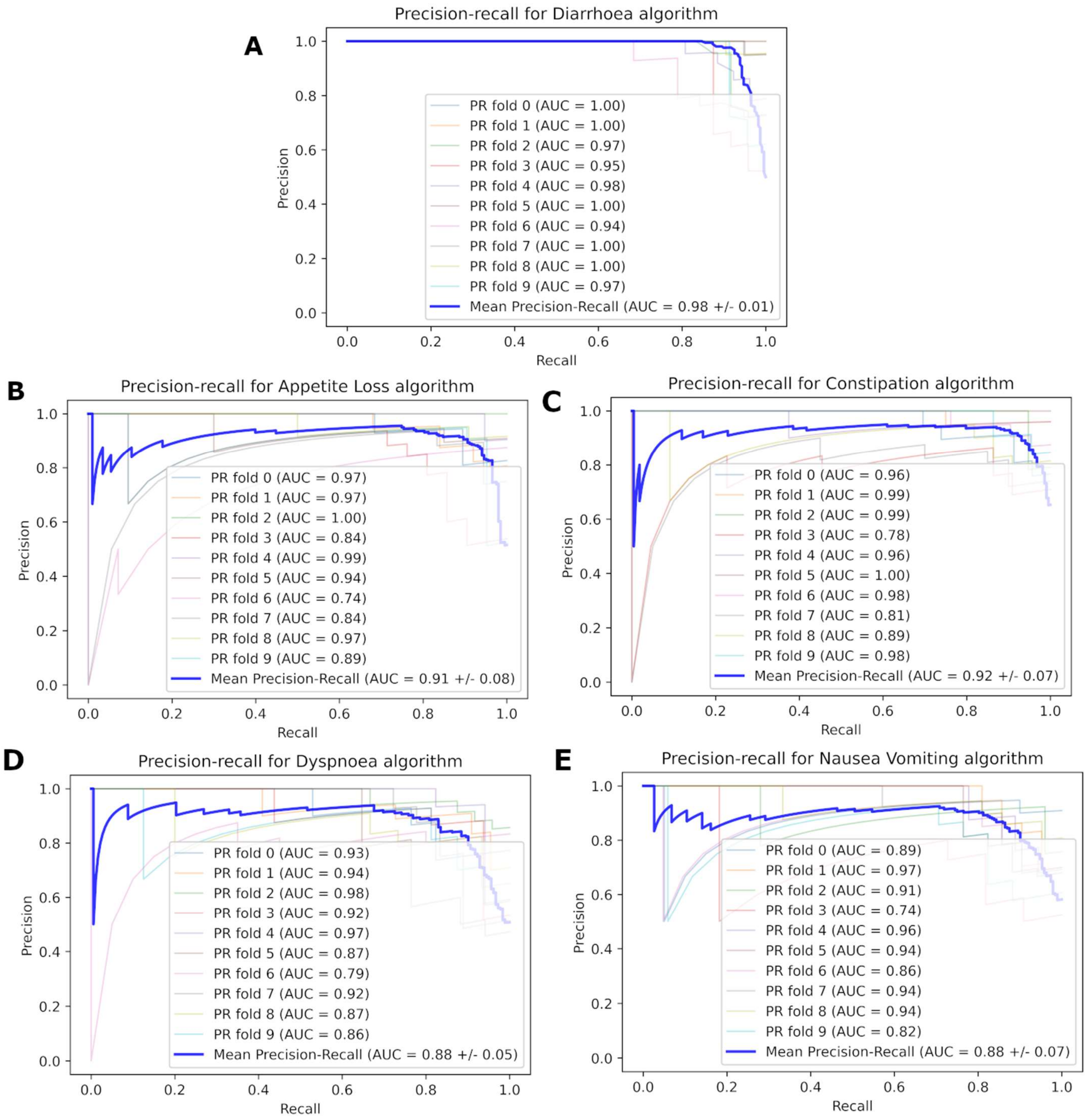

Supplement: S1 File — (PDF) [file pone.0267931.s001.pdf]
